# Update on Biology of Cutaneous T-Cell Lymphoma

**DOI:** 10.3389/fonc.2020.00765

**Published:** 2020-05-12

**Authors:** Zaw H. Phyo, Satish Shanbhag, Sima Rozati

**Affiliations:** ^1^Johns Hopkins University School of Medicine, Baltimore, MD, United States; ^2^Departments of Oncology and Medicine, Johns Hopkins University, Baltimore, MD, United States; ^3^Department of Dermatology, Johns Hopkins University, Baltimore, MD, United States

**Keywords:** cutaneous T cell lymphoma (CTCL), mycosis fungoides, sezary syndrome, single cell profiling, precision medicine, next generation sequencing, tumor microenvironment

## Abstract

Cutaneous T cell lymphomas (CTCL) comprise of a heterogeneous group of non-Hodgkin lymphomas derived from skin-homing T cells. Variation in clinical presentation and lack of definitive molecular markers make diagnosis especially challenging. The biology of CTCL remains elusive and clear links between genetic aberrations and epigenetic modifications that would result in clonal T cell expansion have not yet been identified. Nevertheless, in recent years, next generation sequencing (NGS) has enabled a much deeper understanding of the genomic landscape of CTCL by uncovering aberrant genetic pathways and epigenetic dysregulations. Additionally, single cell profiling is rapidly advancing our understanding of patients-specific tumor landscape and its interaction with the surrounding microenvironment. These studies have paved the road for future investigations that will explore the functional relevance of genetic alterations in the progression of disease. The ultimate goal of elucidating the pathogenesis of CTCL is to establish effective therapeutic targets with more durable clinical response and treat relapsing and refractory CTCL.

## Introduction

Cutaneous T cell lymphomas (CTCL) are a heterogeneous group of lymphoid malignancies derived from skin-homing T cells. The incidence rate of CTCL is 6.4 per million persons and has experienced increases in recent years in part due to better diagnosis (1). Highest incidence rates are seen among African Americans and older individuals, with a four-fold increase in incidence of patients over 70 (2, 3). Mycosis fungoides (MF) is the most common form of CTCL characterized by skin-homing CD4+ T cells. Sezary syndrome (SS) is an aggressive CTCL variant with varying levels of clonal lymphocytes in the blood. MF and SS together account for more than 50% of all CTCL cases (4). Diagnosis of CTCL is challenging due to the polymorphic nature of disease presentation, and a lack of a single definitive diagnostic procedure. Management of limited-stage MF involves more conservative approaches such as skin directed therapies while advanced-stage MF/SS patients are often treated with targeted and systemic chemotherapies, which are often short-lived and associated with adverse reactions (1). Large cell transformation (LCT) is a complication found in advanced-stage MF/SS that is histologically defined by the presence of large, atypical lymphocytes exceeding 25% of lymphoid infiltrate and is historically associated with significantly lower median survival compared to non-transformed MF (5). In addition, expression of CD30 antigen does not manifest in significant prognostic significant differences (6). However, brentuximab vedotin, a CD30 targeting antibody-drug conjugate, has demonstrated significant clinical response in variable CD30^+^ CTCL patients (7, 8).

Beyond differences in clinical presentation, the biology of malignant T cells in MF and SS are thought to be at least in part distinct due to expression of cell surface markers consistent with skin resident effector memory T cells and central memory T cells, respectively (9). However, recent single-cell profiling studies reveal phenotypic plasticity and tumor heterogeneity, suggesting that MF and SS may belong to the same disease spectrum. The molecular and cellular biology of this spectrum of malignancies has yet to be fully decoded. In this review, we will discuss advances in understanding the genomic landscape of CTCL with emphasis on recent NGS studies in further elucidating the pathogenesis of MF and SS. We acknowledge that the fast pace of evolving technologies, such as single-cell profiling, will provide further insights to patient-specific tumor biology.

## Origin of Malignant T Cells

### External Risk Factors

Despite isolated case series of familial mycosis fungoides (10) and links to potential HLA alleles (11, 12), there is no concrete evidence for genetic predisposition. Additionally, infectious agents such as viruses, viral particles (13–15), environmental, geographical (16) or occupational exposure (17) have been contemplated to be triggers for the rise of CTCL but a strong link has not been identified.

Bacterial infections, particularly with staphylococcus aureus, are frequently noted in patients with CTCL and antibiotic therapy usually results in clinical improvement (18–20) Housing mice with CTCL-like features in germ-free conditions led to attenuated tumor burden which was reversible when co-housed with conventional mice (21). A recent prospective study examining 8 patients with advanced-stage CTCL found that transient antibiotic treatment is associated with a decrease in fraction of neoplastic T cells, decreased cell proliferation and a decreased in STAT3 signaling (22). While the mechanistic role is unclear, these data indicate that the commensal microbiome may play a role in malignant T cell transformation and consequently may serve as a therapeutic target.

### Tumor Microenvironment and Dysregulated Cytokine Signaling

T cell receptors (TCRs) recognize and bind to a specific antigen presented by the major histocompatibility complex molecule (MHC); this interaction induces a cascade of phosphorylation and gene expression that results in T cell survival and proliferation. The hyperactivity of the TCR pathway, either by genetic alteration (23–25) and/or continuous contact with antigen presenting cells (26, 27) can result in robust proliferation and continuous activation of T cells leading to disease progression. Malignant T cell clones in MF can be traced to peripheral blood by TCR sequencing and the heterogeneity of malignant T cells between skin lesions might be partially attributed to variation in seeding patterns by peripheral blood (28). The growth and viability of the CTCL cell is partly dependent on direct contact with immature dendritic cells (DC), through interaction between CD40 located on DC and CD40 ligand located on CTCL cells (26). Macrophages and mast cells are also investigated for the roles they play in the tumor microenvironment. *In vivo* mouse studies demonstrated slower rates of progression of human CTCL tumor cells in mice depleted of mast cells (29) and macrophages (30). The malignant T cells also facilitate shaping the tumor microenvironment that is supportive of disease progression. Multiple ligand/receptor interactions, including VEGF/VEGFR (31) and CXCR4/CXCL12 (32), have been characterized for their role in development of a vascular niche conducive to growth of neoplastic T cells. Further research is needed for potential utilization of these vascular niche factors in improving diagnosis and targeted anti-angiogenic therapy. Malignant T cells also secrete galectin-1 and−3, which have been linked to decreased skin barrier function and uncontrolled epidermal proliferation (33), which explains the increased incidence of bacterial skin infections observed in CTCL patients. The functional state of T cell is crucial in the dynamic state of tumor microenvironment. In cancer, T cells operate in a chronic inflammatory state and ultimately enter a hypo-responsive state called T cell exhaustion which is in part characterized by expression of inhibitory receptors (34). Indeed, malignant T cells derived from patients across all CTCL stages display increased expression of inhibitory receptors including PD-1 (35–37), CTLA-4 (38), and LAG-3 (37). The role of inhibitory receptors in T cell exhaustion implies that they can be targeted to effectively reinvigorate effector T cells. Nivolumab (anti-PD-1) was found to be well-tolerated patients with relapsed or refractory hematologic malignancy, which included patients with MF (39). More recently, a multicenter phase II trial of pembrolizumab (anti-PD-L1) led to favorable outcomes in patients with advanced MF or SS (40). In 2018, the FDA approved Mogamulizumab (anti-CTLA-4) for treatment of relapsed and refractory MF and SS, after a randomized, multicenter phase III clinical trial revealed superior investigator-assessed progression-free survival compared to vorinostat (41).

Investigation of the role of cytokine profile in CTCL stemmed from the observation that atopic dermatitis, a classically Th2-skewed disease, is more prevalent in family history of MF patients (42). PBMCs from SS patients of various stages revealed decreased IL-4, IL-2, and IFN-γ, suggesting that malignant T cells in CTCL resemble the cytokine profile found in Th2 cells (43). Th1 pattern, found to be prevalent in early stage of the disease, may allow antitumor response to local disease. In later stage, there is Th2 and Th17 bias with global depression in cytokine expression, which may signify loss of immune function and T cell exhaustion (44). Gata-3 and JunB, Th2 cells-specific transcription factors, are expressed starting in early disease (45). Induction of Th2-dominant biology is partially linked to expression of extracellular matrix proteins periostin and thymic stromal lymphopoietin (TSLP) by dermal fibroblasts, which subsequently activates release of Th2-specific cytokines in CTCL cells (46).

The immune responses that dictate CTCL progression or inhibition are largely unknown and our understanding is complicated by conflicting results in the literature. For example, pro-inflammatory responses, such as Th17 are thought to promote tumor progression and limit anti-cancer Th1 response (47). Recently, a few case series have shown that TNF-inhibitors and IL-17 inhibitors promoted the development or progression of MF in patients with inflammatory bowel disease, rheumatoid arthritis or misdiagnosed psoriasis (48). Contrary to previous reports, these results suggest that inhibition of Th17 mediated immune responses lead to CTCL disease progression.

On the other hand, regulatory T cells (Tregs) have been associated with Sezary syndrome and are thought to be an indicator of poor outcome (49). However, a recent single-cell profiling study of CTCL identified Treg transcription factor Foxp3 as the strongest predictor of early rather than late-stage Sezary syndrome (50). These data indicate that tumor FoxP3 expression may suppress CTCL disease rather than promote progression as previously thought. Therefore, it is crucial to investigate the factors that drive Th17 and Treg immunity in CTCL to better understand the mechanisms that affect disease outcome.

Our current knowledge on CTCL immunophenotype, cytokine profile and its interactions within the host immune system denote an intricate tumor microenvironment and present numerous potential targets for therapy.

## Genomic Landscape of CTCL

### Genetic Aberrations

In the past few years, multiple groups have applied deep sequencing techniques including whole genome and whole exome sequencing to explore the genomic landscape alterations in cutaneous lymphoma (23–25, 51–53). These results have broadened our horizon in the understanding of the pathogenesis of this heterogeneous group of malignancies by identification of new somatic mutations, and common mutagenic pathways.

TP53 is a tumor suppressor, which responds to DNA damage and other stress signals and is often dysregulated in cancer (54). While a unifying oncogenic driver is absent, TP53 is a notable tumor suppressor in CTCL with a somatic mutation (23) and gene deletion (24, 25) on chromosome 17p detected in 19 and 37% of studied CTCL patients, respectively (55). However, TP53 mutation status does not endow any changes in prognosis in primary SS patients (56). The constitutive activation of nuclear factor kappa B (NF-κB) pathway, located downstream of TCR signaling, has been implicated to play a key role in tumor resistance to apoptosis in CTCL (57). Recent genomic sequencing by multiple groups have reported on alterations in PLCG1 (58), CARD11 (25), TNFRSF1B (23) and KIT (55) that are involved in NF-κB pathway. These alterations are involved in regulating T cell survival and proliferation and/or control transcriptional programs downstream of key T cell signaling. The involvement of the NF-κB pathway served as a rationale for the potential use of bortezomib, an inhibitor of NF-κB signaling, which exhibited 67% overall response rate with acceptable drug tolerability in a phase II clinical trial (59).

To address the low incidence rate of CTCL and small patient cohorts with variations in geographic or subtype origin found in most NGS studies, Chang et al. created an integrated CTCL genomic dataset by collecting and re-analyzing raw genomic data of 139 patients with CTCL from seven different sequencing studies of MF/SS (55). Consolidation of previous NGS cohorts improved statistical power and revealed insights to specific patterns of genetic aberrations. TP53 mutations were found to be mutually exclusive from NF-κB pathway gene mutations, indicating that tumor variants might arise from distinct genetic backgrounds. Moreover, mutual exclusivity was observed within the NF-κB pathway genes, suggesting that CTCL tumorigenesis may be triggered by one pathway alone. Cases that did not carry p53 or NF-κB mutational changes did not have any other significant abnormalities, indicating that other important changes in the transcriptome or epigenome may have a major role in tumorigenesis.

In addition to the NF-κB pathway, the JAK3/STAT3 signal transduction pathway is also well-characterized for its role in survival and proliferation of malignant T cells (60–62). Genomic studies have shown that gain of function point mutations and copy number gains in this pathway are frequent and correlate with gene expression of STAT3 and increased expression of pro-inflammatory cytokines IL17 and IL22, downstream targets of STAT3 activation that likely play a role in tumor progression (25, 63). In a mouse model of CTCL, transgenic STAT3 hyper-activation in T cells was linked to IL-17 and IL-22 expression and phenotypic features of CTCL (21, 64). Staphylococcal enterotoxin A (SEA) has been shown to drive IL-17 expression through a JAK3/STAT3-dependent pathway in malignant T cells when co-cultured with non-malignant T cells, suggesting that SEA-driven cross talk between malignant and non-malignant T cells are needed for oncogenic activation of STAT3 (64).

Beyond somatic mutations characterized previously, genes may also be amplified or deleted due to somatic copy number variants (SCNVs). Compared to other cancers, CTCL is unique in that it harbors a disproportionately high number of SCNV compared to somatic mutations (24). In addition to 17p deletion involving TP53, other tumor suppressors such as RB1, PTEN and CDKN1B have been reported, along with amplification of STAT3 (17q) and MYC (8q) (23–25, 65–67).

### Epigenetics

Epigenetic abnormalities have been recognized for their role in altering gene expression of oncogenes and tumor suppressors and ultimately contributing to malignant cell transformation in cancer (68). Both hypo-methylation and hyper-methylation signatures have been observed in CTCL. DNMT3A, a gene encoding a methyltransferase, is often mutated or deleted in CTCL (55), signifying that genetic aberrations may underlie epigenetic dysregulation. The association between DNMT3A and mutated genes highlights the importance of integrating findings from sequencing studies and epigenetic findings. Histone deactylases (HDACs) remodel chromatin architecture by removing acetyl groups from histones and have been characterized as a therapeutic target in cancer (69). Vorinostat (70) and Romidepsin (71) inhibit HDACs which leads to gene expression of cell cycle regulators and promotes tumor cell apoptosis.

MiRNAs are non-coding RNA involved in epigenetic mechanisms that are implicated in essential cellular processes (72). The miRNA expression profile has been investigated through different CTCL populations. Increased expression of miR-213, miR-486, and miR-21 were proposed to promote apoptotic resistance in CTCL cell lines (73). Notably, multiple groups have identified miR-155 for its role in pathogenesis of MF (74–76). Later, a causal link was established between JAK/STAT signaling and expression of miR-155 and its host gene BIC (B cell integration cluster), implying that STAT5/BIC/miR-155 can be targeted for therapy (77). MiR-155 inhibitors have been assessed in phase I-II clinical trials for their safety, tolerability and clinical activity with encouraging results (78, 79). MiRNAs may also play a role in predicting prognosis, with multiple groups demonstrating that a panel of miRNAs could be used to effectively stratify patients based on prognosis (80, 81). These types of prognostic markers must be validated in large multi-centered, ideally prospective cohort, studies. While many miRNAs have been implicated in CTCL, further research is needed to delineate the mechanisms in which miRNAs are deregulated and how it impacts disease progression.

Emerging technologies, such as transcript—index ATAC-seq allows researchers to interrogate epigenetic signatures indexed by TCR sequence-based T cell clonality, further refining single-cell resolution in dissecting tumor heterogeneity of CTCL (82).

### Emerging Frontiers in CTCL

Emerging technologies in NGS allows researchers to interrogate the DNA sequence and transcriptomes of tumors at the resolution of single cells and has provided an unprecedented view of cellular processes. These advances in technology will rapidly evolve our understanding of tumor transformation and progression.

Single cell RNA-sequencing provides an in-depth view of gene expression profile of each tumor cell as well as an insightful perspective of major cellular components in relation to the tumor microenvironment. The heterogeneity of tumors between patients with CTCL has been well-documented at a clinical and molecular level in the literature. This knowledge has been further cemented by striking distinct gene expression profiles seen in advanced CTCL patients (83). Nevertheless, Gaydosik et al. also identified a 17-gene expression signature that was common between highly proliferative tumor cells in all samples. Interestingly, these signatures overlap with expression of TOX, a previously reported marker for identifying malignant lymphocytes in CTCL (83). Others have utilized single-cell sequencing in conjunction with artificial intelligence (AI)-based learning to create a framework that broadens the clinical applicability of their results in CTCL (50).

Using a single-cell flow cytometry-based assay, Buss et al. isolated malignant cells from 8 treatment-naive patients with SS and assessed the expression of 240 surface antigens and single-cell RNA sequencing for 110 T-cell-relevant genes. Based on surface antigen expression, malignant T cells were divided into distinct subpopulations and exhibited different sensitivities to HDACi treatment (84). The presence of multiple subpopulations with variable sensitivity to a single agent lends further support for the need for combination treatments that are informed by the patient's unique malignant clonal characteristics. The synergistic epigenetic-modulatory effect of histone acetylation of DNA demethylation results in global CpG methylation alterations as well as reexpression of tumor suppressor genes that was not achieved by single agent treatments (85). Preclinical and clinical investigations have demonstrated the combinatorial use of different epigenetic modulators together or in combination with other treatments (85–90).

Mutual exclusivity in CTCL reported by Chang et al. (55) as well as single-cell RNA sequencing analysis may contribute to the basis for molecular subtyping of the disease similar to other hematologic malignancies such as systemic diffuse large B cell lymphoma (DLBCL) (91) or acute leukemia (92). Combination approaches in therapy targeting multiple signaling pathways and clonal subpopulations can lead to unprecedented improvements in survival and quality of life.

Emerging technologies are further refining single-cell resolution in dissecting tumor heterogeneity of CTCL. Applications of these technologies could enable novel therapies or treatment strategies that have previously been deemed unlikely in CTCL. One such example is chimeric antigen receptor (CAR) T cell therapy, which primes the patient's own T cells to activate upon recognition of a tumor-specific antigen (93). CAR T therapies targeting CD19 in B cell malignancies led to durable remissions for refractory B-ALL (94) and DLBCL (95) patients. In contrast, immunophenotypic methods yield high overlap in surface markers between malignant and normal T cells, which presents a major challenge in targeting cancerous cells. Future studies could reveal more subtle phenotypic differences in T cells as NGS technologies enables higher resolution of clonotypic T cells.

## Conclusion

We have yet to identify a major oncogenic driver or a constellation of genetic and epigenetic alterations that lead to malignant clonal T cell expansion seen in CTCL patients. However, recent NGS data including single cell sequencing have identified genetic aberrations in major signaling pathways and epigenetic components that play an important role in pathogenesis of CTCL. They have provided biomarker signatures that could be utilized in the future to identify early disease, predict disease progression, and tailor treatments to individual patients. It is important to ask the right type of research questions when performing such studies to extract relevant data and improve clinical outcomes. Further understanding of tumor biology in CTCL is imperative in developing patient-specific treatment with minimal side effects, a cornerstone of precision medicine. We are entering a new area of discovery that will optimize our management for this heterogeneous group of malignancies.

## Author Contributions

ZP and SR performed literature search and wrote most parts of the manuscript. SS performed literature search and wrote some parts of the manuscript. SR conceived the framework of this review article, provided insights, and edited the manuscript.

## Conflict of Interest

The authors declare that the research was conducted in the absence of any commercial or financial relationships that could be construed as a potential conflict of interest.
